# Exploring Youth Perspectives on Digital Mental Health Platforms: Qualitative Descriptive Study

**DOI:** 10.2196/69907

**Published:** 2025-05-29

**Authors:** Sarah Daniel, Lauren Volcko, Emilie Bassi, Julia Hews-Girard, Katherine Bright, Marianne Barker, Lia Norman, Karina Pintson, Geneca Henry, Sumaya Soufi, Chukwudumbiri Efrem Omorotionmwan, Melanie Fersovitch, Leanne Stamp, Karen Moskovic, David W Johnson, Gina Dimitropoulos

**Affiliations:** 1 Faculty of Social Work, University of Calgary, MacKimmie Tower, 2500 University Dr NW, Calgary, AB, T2N 1N4, Canada, 1 4032205942; 2 Alberta Children's Hospital Research Institute, Calgary, AB, Canada; 3 Faculty of Nursing, University of Calgary, Calgary, AB, Canada; 4 Mathison Centre for Mental Health Research and Education, Calgary, AB, Canada; 5 Hotchkiss Brain Institute, Calgary, AB, Canada; 6 School of Nursing and Midwifery, Mount Royal University, Calgary, AB, Canada; 7 School of Nursing, Thompson Rivers University, Kamloops, BC, Canada; 8 Faculty of Rehabilitation Medicine, University of Alberta, Edmonton, AB, Canada; 9 Heroes in Mind, Advocacy and Research Consortium, Edmonton, AB, Canada; 10 Recovery Alberta, Women’s Mental Health Clinic, Edmonton, AB, Canada; 11 Alberta Health Services, Edmonton, AB, Canada; 12 Department of Pediatrics, Cumming School of Medicine, Calgary, AB, Canada

**Keywords:** digital mental health, youth mental health, youth perspectives, engagement and retention, trust and privacy, personalization

## Abstract

**Background:**

The increasing prevalence of mental health disorders among youth underscores the need for accessible and effective interventions. Digital mental health (dMH) platforms like Innowell offer promising solutions by increasing access to mental health care for young people. Innowell is a web-based platform that supports youth mental health by providing personalized measurement-based care in collaboration with a youth’s health care providers. However, understanding youth perspectives on these platforms is crucial for ensuring successful implementation and sustained engagement.

**Objective:**

This study aimed to explore youth perspectives on the implementation of the Innowell platform, identifying key factors influencing uptake, engagement, and long-term retention.

**Methods:**

A qualitative descriptive approach was used to examine youth perspectives. Data were collected through 9 focus groups and 1 interview, involving 39 participants aged 15‐24 years from urban (23/39, 59%) and rural (16/39, 41%) communities in Alberta, Canada. Participants were recruited through mental health clinics and community organizations. Thematic analysis was conducted on the transcripts to identify factors that support or hinder engagement with the platform.

**Results:**

Participants emphasized the importance of privacy, security, and personalization in building trust in the platform, with 72% (28/39) reporting that clear communication about data protection would increase their likelihood of use. Progress tracking features, such as symptom trend visualizations and diaries, were identified by 65% (25/39) of participants as critical for sustaining engagement. Ease of use was highlighted, with 58% (23/39) preferring mobile app functionality over web-based interfaces. Dynamic content and personalized notifications were suggested as strategies to maintain long-term use, with 64% (25/39) of participants valuing customizable reminders to encourage daily interactions. Rural participants (16/39, 41%) noted the need for offline functionality due to inconsistent internet access. In addition, participants recommended features such as crisis support, professional communication channels, and access to local mental health resources.

**Conclusions:**

Youth-centered design is essential for enhancing the usability and engagement of dMH platforms like Innowell. Key features prioritized by participants included privacy, security, progress tracking, and personalization. Dynamic and user-friendly interfaces, along with the ability to customize notifications and access professional support, were critical for fostering long-term engagement. Insights from this study provide actionable recommendations for optimizing dMH platforms to meet the mental health needs of young people, particularly in diverse urban and rural settings. Future research should explore implementation strategies tailored to specific user demographics to enhance the scalability and impact of dMH interventions.

## Introduction

The number of youth and young adults affected by mental health disorders has increased in the last 10 years [1,2]. Adolescence and early adulthood, ages 12‐24 years [3], is a peak period of vulnerability for mental ill-health, with 62.5% of mental health disorders emerging before the age of 25 [4]. Mental health conditions of concern include depression, anxiety, eating disorders, bipolar disorder, and substance abuse disorders among others [1,2,4]. Globally, mental health disorders are the leading cause of disability in youth and are the sixth leading cause of disability adjusted life years [5]. The number of youths underdiagnosed or undertreated remains a significant global issue due to barriers to accessing youth mental health services [6,7]. This involves social stigma, limited awareness of available mental health resources [7], and concerns about the therapeutic relationship, including confidentiality and privacy [8,9].

Digital mental health (dMH) has the potential to address the treatment gap of those who do not receive services and increase accessibility of mental health care to youth and young adults [10,11]. The dMH includes both mobile apps and web-based platforms [12] that can augment in-person and traditional mental health services. Youth tend to be more familiar with the digital world and use the internet at progressively younger ages [13]. Given that social interactions and educational commitments are increasingly taking place through digital modes, youth are becoming more inclined to seek mental health support through the internet [14-16]. Research suggests that stigmatizing beliefs surrounding mental illness drive many youth to self-initiate their mental health care by accessing the internet for educational information or assistance regarding mental health concerns, because it is perceived to be more confidential and private than face-to-face services [17]. Of youth aged 13‐17, approximately 22.2% use internet-based services to seek information regarding behavioral issues [17]. Therefore, there is a growing need for youth-oriented dMH platforms to address some of the challenges of traditional face-to-face mental health care [18,19].

Youth and young adult perspectives on dMH platforms are critical to facilitate retention and uptake of these platforms [20]. Including youth perspectives in dMH research ensures that platforms are accessible, relevant and align with their unique needs and experiences [20,21]. A qualitative study conducted by McKenna et al [22] found that youth valued measurement-based care when embedded in a digital platform and used in their sessions with their therapists because it helped them track their health information between appointments. Generally, dMH platforms have been noted by youth to increase accessibility to mental health care [23], however, the information presented on these platforms must be easy to understand [23]. Youth also noted that dMH may aid self-determination and promote anonymity when asking for mental health support [23].

Despite the benefits of dMH for youth, knowledge regarding the reasons youth use and continue to engage with these platforms is limited [24]. To date, research suggests that reasons for lack of engagement in dMH platforms include limited in-person elements, for example, face-to-face support from a parent, peer or professional [13], privacy concerns [25], technical difficulties [25], and inadequate personalization of the platform [25,26].

A 2019 systematic review and meta-analysis assessing the effectiveness of dMH platforms for youth with anxiety and depression revealed that out of the 34 identified interventions reported, none involved young people in the design process [27]. In contrast, a 2020 review identified 30 platforms that did involve youth design [28]; however, the impact of this has not yet been fully explored as few are widely available for use [29]. One example of a codesigned, personalized, and youth-centered dMH platform that is available in Australia and Canada is Innowell [10,30-32]. The perspectives of youth on the facilitators and challenges in using dMH to access mental health resources and as an adjunct to mental health interventions are scarce.

This study seeks to ascertain the perspectives of youth and young adults regarding the potential barriers and facilitators of using dMH. Understanding what youth expect from a dMH platform can inform recommendations for implementation. To that end, this project asked the following research question: what influences youth engagement with and sustained retention of digital mental health platforms?

## Methods

### Ethical Considerations

This study is part of a larger multimethod research project aimed at investigating the implementation of a measurement-based care dMH platform for Albertan youth and young adults and was approved by the University of Calgary’s Research Ethics Board (REB20-1137) [30,31]. Informed consent was obtained from all participants, and participants under 18 years were required to complete a decision-making capacity assessment to not require parental consent. Participants received instruction to avoid disclosing identifying information, and during transcription any direct identifiers (names, cities, and workplaces) were removed to protect participants’ privacy. Focus group participants were compensated for their time at a rate of CAD $25 (US $18.07) per hour.

### Recruitment

Recruitment occurred from November 2020 to June 2022. Participants were recruited using self-selected sampling, where participating communities and Innowell testing sites displayed and shared study recruitment posters. Project Leads coordinated with leaders in partnering communities to display the recruitment poster in mental health clinics and community spaces. In some cases, clinicians discussed the opportunity to participate in the study with their clients aged 15‐24 years. Interested youth then emailed the study coordinator to express their interest in participating. Inclusion criteria for participants required being between the ages of 15 to 24 years, proficient in written and spoken English, residing in Alberta, and finally, able to use web-based technologies on a computer, tablet, or smartphone. Interested participants were emailed the date and time of the focus group, along with a link to complete the informed consent form and a brief demographics survey to assess participant characteristics. In accordance with the research ethics protocol, a decision-making capacity assessment was administered to participants under the age of 18 years. A perfect score of 4 out of 4 was required to be determined a mature minor, for which parental consent would not be required. If a participant scored lower than 4/4, the missed items were reviewed and retested with a limit of 3 total attempts. Participants without a perfect score after 3 trials were determined to not be mature minors and were ineligible to participate without parental consent. Once the completed consent form was received, the participants were sent a link to join the focus group via Zoom Video Conferencing Software.

### Data Collection

Focus groups were conducted as the primary source of data collection. One interview was conducted at the request of the participant. A semistructured interview guide was created to focus on general thoughts and opinions on the Innowell platform and what would support or hinder youth from using it in their community. All focus groups and interviews were facilitated virtually via Zoom Video Conferencing software, audio recorded, and transcribed verbatim by a professional transcriptionist [30,31]. Each focus group lasted up to 90 minutes in length and included 2 to 8 participants. The focus groups and interview included one facilitator and 1 youth research partner note-taker or chat moderator. Participants were first introduced to Innowell through a short video and demonstration of the platform before the focus group discussion. In total, 9 focus groups and one interview were conducted with 39 participants (n=39). Participants represented both urban and rural communities across the province of Alberta, Canada. Sample characteristics are in Table 1.

**Table 1.** Demographic characteristics of the participants (N=39) were analyzed.^a^^b^

		Values
Sex, n (%)		
	Female	25 (64.1)
	Male	7 (17.9)
	Missing	7 (17.9)
Sexual orientation, n (%)		
	Heterosexual	25 (64.1)
	LGBTQIA2S+	10 (25.6)
	Missing	4 (10.2)
Age, n (%)		
	15‐17 years	21 (53.8)
	18‐24 years	16 (41.0)
	Missing	1 (2.5)
Ethnicity^c^, n		
	Other North American origins	7
	European origins	21
	Asian origins	5
	Other	6
Student or employment status, n (%)		
	Employed	22 (56.4)
	Student	29 (74.3)

a “Other” includes sociodemographic information for fewer than five participants to protect anonymity.

b Missing includes “prefer not to answer“ and “missing” responses. These are combined for clarity and confidentiality.

c Values for ethnicity do not equal 100% as participants were allowed to select multiple responses.

### Data Analysis

This study used a qualitative descriptive methodology to thoroughly capture the thoughts, perspectives, and experiences of participants who have experiences in health care settings [33,34]. This allowed for the exploration of youth perceptions of challenges and enablers of dMH implementation that are grounded in their lived-experiences of navigating mental health within their communities.

The qualitative analysis of the focus group and interview data was conducted using a 6-stage thematic analysis process: familiarization with the data, generating codes, constructing themes, revising themes, defining themes, and producing the report [30,31,35]. This process followed an iterative codebook approach, allowing for the development of consistency with the generation of codes across focus groups [35]. Three young adult researchers familiarized themselves with the transcripts. They then independently created preliminary codes with support and oversight from the qualitative lead. After coding, they exchanged transcripts and collaborated to resolve any discrepancies, ensuring the validity of the codes through consensus. The coders and the research team then developed themes from the codes. The team engaged in group discussions about the themes, actively documenting their insights to maintain a rigorous and transparent process. Following these 6 steps enhanced the accuracy and credibility of the analysis [30,31]. The sample of 9 focus groups and 1 interview was deemed sufficient based on the concept of “information power,” which suggests a qualitative sample’s quality relies on its richness, relevance, and depth rather than the number of participants. This approach ensures ample information was gathered to draw meaningful conclusions from the content of the data [30,31,36].

### Research Team Positionality

Our team comprises qualitative leads with extensive experience in both research and frontline clinical support, bringing a wealth of knowledge about the complexities of health care settings and a deep understanding of how to effectively support youth. The study team also included youth and young adult coresearchers throughout the entirety of the study, including recruitment, data collection and analysis, and writing the research findings. Youth coresearchers contributed to the research process and offered guidance and knowledge in engaging youth participants. The study team also focused on the power dynamics inherent in conducting focus groups with youth. Recognizing these dynamics, the study team prioritized creating a safe and supportive environment by including a youth coresearcher in every session. The study team also actively encouraged youth to choose their level of engagement, including whether to keep their videos on or off or use the chat, ensuring that their comfort and agency were prioritized in the research process. Through these strategies, we aimed to foster a more equitable dialogue and enhance the validity of our findings.

### The Innowell Platform

The dMH platform, Innowell, is an Australian web-based tool youth use in conjunction with their mental health providers [37-39]. Specifics regarding the Innowell platform are described elsewhere [30-32,38,39]. Previous work from the research group has explored the implementation of this platform in school settings, mental health providers’ perceptions of using and engaging dMH with youth, and the creation process of an implementation protocol for using dMH in stratified care.

## Results

### Demographics Summary

The study included participants from various communities across Alberta (withheld to protect anonymity). Participants were aged 15‐17 years (n=21) and 18‐24 years (n=16), with one missing response for age. Regarding sex, 7 participants identified as male, 25 as female, and 6 did not report. Sexual orientation was diverse, with most participants identifying as straight (n=25) and 10 as Lesbian, Gay, Bisexual, Queer or Questioning, Intersex, Asexual, 2-Spirited, and other orientations (LGBQIA2S+), combined to protect anonymity. Ethnic backgrounds included European origins (n=21), Other North American (n=7), Asian origins (n=5), six as other or unsure. Regarding employment or student status, 22 were employed and 29 were students. For a full overview of the demographics, see Table 1.

### Theme 1: Building Trust—A Digital Safe Space

#### Confidentiality, Privacy and Data Security

Participants in the study agreed that youth valued and trusted a dMH platform that would grant them the “comfort of …confidentiality and being up front and honest*,*” (FG2). By improving confidentiality and privacy, dMH enables a safe space for youth disclosure of mental health challenges and requests for support. A highly secure and confidential dMH platform would enable youth to access mental health support by reducing the stigma associated with seeking in-person mental health care. One participant stated:

*There’s always that stigma around mental health …[by] having it so that you don’t have to be right in-person with the doctor is just a lot easier for people to maintain confidentiality and to be more expressive.*

[FG6]

Participants across all focus groups emphasized a deciding factor in their use of dMH would be the strength of data privacy and security features determining how their information is used and stored. As illustrated by one participant, “You have to remind the people that are using this (dMH) that you’re not collecting their data” (FG9). Another participant indicated, “I would want to know who can view the information, where the information is going etc,” (FG4). To build trust with youth, participants strongly advocated that dMH must clearly state how user data is used and protected. Factors that contributed to building trust in dMH security measures included the appearance and professionalism of the platform. Participants identified that commercial advertisements decreased the platform’s appeal, with one stating:

I would be hesitant to download that app knowing that I could still be targeted with ads or that information could be leaked to third parties and trying to sell me something with it.

[FG9]

The dMH was seen as having the potential to build safe spaces for youth disclosure if “you have that comfort of some sort of confidentiality and being upfront and honest*”* (FG2) when providers are onboarding youth to use dMH in their care.

#### Keeping “Control Over the Situation”: Access to Information and Involvement of Others

Participants frequently reported that youth must have jurisdiction over who accesses their personal information on dMH, including providers and parents or guardians. Many participants advocated for reassurance that their information was “protected, nobody else is going to see [their] information [and] it’s not going to be shared with anybody outside of [their] clinician and [themselves] or [their] support person” (FG6).

To facilitate this level of privacy, some participants identified “certain healthcare providers that [they] would not want involved in [their] mental health care,” and suggested that youth are “able to pick the adults that they were going to be disclosing information to” on dMH platforms (FG1). All participants noted that youth must have control over the level of parent or guardian involvement in dMH. In fact, some participants who reported having a conflictual relationship with their parent or guardian were less inclined to use dMH if family members had any involvement in their care. Overall, youth wanted “control over the situation” to defend their confidentiality and privacy: “...if someone is being added they know they can say, ‘Hey, I don’t want this person here’” (FG6).

### Theme 2: Platform Features to Maximize Youth Engagement

#### Onboarding or Getting Started With dMH

The participants agreed that youth must be onboarded to dMH to increase digital literacy and trust and generate enthusiasm for dMH’s utility in their care. Once receptive to the idea of dMH, participants expressed a preference to receive collaborative onboarding by a provider or dMH expert. One participant imagined receiving a “thorough” walk-through in which they receive a “step-by-step explanation of what you would do [led by] the professionals behind the app” (FG4). Other participants emphasized the need to understand the purpose and the features of dMH to increase the likelihood of engaging with dMH applications. Participants suggested that the onboarding process to dMH be educational, informing youth of “the why behind using it [as] people lose interest when there isn’t much detail explained” (FG4).

#### Progress Tracking

Participants frequently reported the value of tracking their mental health within dMH, including visualizing trends through graphs. One participant noted:

*I know for myself, I’m really interested in data and trends about myself and my health and being able to broaden that to my mental health is kind of a cool way to introduce it.*

[FG9]

Relatedly, a participant noted that seeing progress through dMH tracking may be motivating when a change in their mental health is not perceptible on a day-to-day basis to them and others in their lives.

*It is about getting better, but it’s not always going to be about seeing this dramatic increase and having this weight lifted off your shoulders. I think being able to document things like, “What happened to me today?” Or “how’s this week been?” Or “how am I feeling or dealing with whatever’s going on?”*

[FG6]

A platform feature allowing youth to store and detail personal information, including symptoms and important goals, can serve as an invaluable tool for tracking progress . One participant expressed, “Maybe there could be areas where you could store things and keep things, maybe make a diary page or something” (FG2). This feature would enable users to document their thoughts, feelings, and experiences over time, facilitating self-reflection and self-awareness. Overall, participants noted that they would use dMH regularly if it served to track and enable reflection on their mental health progress and fluctuations in symptoms.

#### User Experience

Participants throughout the focus groups valued user-friendly design of dMH, including ease of navigation and accessibility. One participant described a facilitator to consistent dMH use as being if “the dashboard itself is easy to understand and navigate” (FG9)*.* Participants expressed a clear preference for an app, or mobile-based, interface compared to a web-based one: “I think it should be an app in the App store because if that is an app in the App Store, it would be accessible to everyone” (FG1)*.* Participants indicated it would increase the chances of discovering dMH, as “youth “looking for meditation [applications] or…games [might] come across it*”* (FG1). In addition, participants saw potential in the dMH interface facilitating access to mental health education and resources. One participant remarked:

*It could give you helpful information to make your mental health better and to give you more activities to help you and places to access phone numbers or places to contact if you need help. That would be amazing.*

[FG1]

Other participants suggested that dMH could direct youth to local mental health resources and crisis hotlines.

The ability to customize the platform’s features and resources to suit a youth’s unique needs was also desirable to participants. A participant suggested users be granted the ability to “edit when notifications are sent, when I can talk to someone, who I can talk to and more would be great so it won’t be stressful and I can run on my own time”(FG2). A personalized dMH could cater to a wider “variety of people and it can connect [them] with the right resources” (FG4). Another suggested utility of dMH included direct communication with a professional, who “could reach out to you through the app. You could maybe have a personal chat area” (FG2). Other participants expressed interest in dMH platforms with specific features supporting youth in crisis.

*I think having emergency functions be very accessible would be important as well. Something like a ‘chat now’ function that’s right on the home screen or close to it so that if someone is in crisis, they can access the help they need*.

This highlights the critical need for in-app support for urgent situations, accessible and emergency functions, complementing the platform’s diverse set of resources, including educational materials, links to available mental health services and crisis hotlines.

### Theme 3: Sustaining Engagement—Strategies for Optimum User Retention

#### Barriers to Consistency: The Challenge of Integration

Across all focus groups, participants identified barriers to integrating dMH into their lives. One participant noted, “For me personally, using apps in the past, after a certain point you just kind of get bored of it” (FG6). Another difficulty was prioritizing dMH in the face of other pressing demands and daily routines: “I think that I’ve had mental health apps in the past, but it’s just remembering to do it and having it as a priority it was really tough….” (FG7). Participants identified competing online priorities such as social media as a barrier to using dMH: “Why would I use a mental health app when I can go on Instagram instead. There’s nothing enticing me to use the app” (FG6), and “Social media gets in the way of using mental health apps sometimes*”* (FG3). Overall, these quotes underscore the necessity for dMH platforms to be engaging, easy to integrate into daily routines, and compelling enough to compete with other online activities to ensure sustained user retention.

#### Suggestions to Reduce Distractions and Maintain Attention

An additional strategy to encourage motivation and continued use of dMH mentioned within the focus groups was the integration of reminder notifications. One participant stated:

*...if I’m getting notified all the time to maybe update or maybe there’s like a quote of the day every day that it’ll send me… I think that would be very useful, and something very appealing to the eye.*

[FG2]

Another participant provided an example of how notifications and prompts promoted reflection and participation with the platform.

*I have the app “Calm” on my phone … you can get a notification at the end of the day asking, “How are you feeling at the end of the day?” Sometimes, a lot of the time I just swipe up and sometimes I actually take the time to think about how I’m actually feeling. It’s nice for the most part.*

[FG3]

However, other participants did not find consistent prompts necessary:

*Some apps I know I have deleted before if I have set the notifications on and they are just constantly sending me notifications to the point where it gets annoying.*

[FG2]

Incorporating timely and personalized notifications could, therefore, play a role in keeping young users engaged and motivated to consistently use dMH platforms, as suggested by one participant.

*If you check your phone, you see the app which reminds you to do your daily check in or maybe a reminder to do it later in the day around lunchtime or afternoon when you accomplish things or have things that affect your mood. I think that would be very important and useful for people.*

[FG2]

The ability for dMH platforms to incorporate additional elements to increase engagement was valued by participants, reflected in a broader sentiment that applications lacking new content tend to be forgotten: “If an app never has anything new on it or different things to try, I find I forget about the app after a while” (FG2). Likewise, one participant noted the power of rewards to attract attention to the dMH platform.

People are very motivated by rewards and attention as well. Because like social media—even though you’re not getting a specific reward, you’re getting attention from others, you’re getting communication and it kind of feeds you … so, I think if either some kind of award or like “Congrats you’ve made it this far! Here’s like a little boost of encouragement,” or something like that. People like that—that positive reinforcement.

[FG6]

Therefore, incorporating timely, personalized notifications can significantly enhance the appeal and retention of dMH platforms, ensuring they remain a valuable and consistently used resource for young users.

## Discussion

### Principal Findings

This study aimed to explore youth perspectives on optimizing the implementation of dMH platforms. Three themes were identified from the inductively coded data. The first theme emphasizes the importance of trust in dMH, protecting youth information and creating a safe digital space. The second theme is the identification of specific platform features to enhance the engagement of youth on the platform. The third theme describes the need for dMH platforms to maintain ongoing engagement of youth by removing obstacles to consistent use of the dMH platform and highlighting strategies to reduce user fatigue and boredom.

### Theme 1: Building Trust—A Digital Safe Space

Our findings suggest that transparency in a dMH’s privacy and confidentiality protections is essential to build youth trust. Other research supports confidentiality’s importance for youth across both in-person and digital mental health care settings [8]. Further supporting our results, youths’ knowledge of a platform’s privacy and security features was identified as a critical factor in increasing engagement with dMH [40]. Despite confidentiality measures contributing to youths’ trust in a dMH platform, a study identified this as insufficient for long-term, sustainable engagement from youth [18].

Intertwined with their emphasis on confidentiality, participants valued the power and autonomy to restrict who can see their mental health information on the dMH platform. Youth most expressed concern about parents and guardians being involved in their dMH care. The findings from our study underscore the critical importance of maintaining youth autonomy and control within dMH platforms, including the ability to selectively involve providers, parents, or guardians. Consistent with our findings, in a preimplementation survey of youth interest in a dMH platform, parental consent was identified as a deterrent for youth participation in dMH [41]. Contrasting to our findings, a study investigating youth attitudes toward parental involvement in dMH reported that youth favored parent access to the dMH patient portal, predicting it would increase accountability and engagement, and enable provider follow-ups [42]. The difference in results may stem from the setting: their study focused on clinical care, where parental involvement is more common, whereas our study was based in community settings, potentially encouraging a greater emphasis on independence and privacy. Our findings suggest that to maximize engagement and effectiveness, dMH platforms should allow users to decide who has access to their data, including the flexibility to invite parents or guardians when it aligns with the youth’s preferences and needs.

In line with our finding that that youth value security and privacy measures on dMH platforms, dMH platforms should have robust data security measures to reinforce confidentiality promises. Supporting our findings, a review article identified confidentiality as a “key ethical concern” for users of all ages on dMH platforms [43]. The emotional and psychological effects of data breaches on victims are long-reaching and can include a loss of confidence in the related technology, acute symptoms of posttraumatic stress disorder, and even learned helplessness [44]. Our participants wanted to be informed about dMH’s security and privacy measures to build trust in the platform. More investigation is necessary, but dMH platforms should maintain user trust by clearly communicating the security features present on the platform.

### Theme 2: Platform Features to Maximize Youth Engagement

Our findings suggest youth prioritize their autonomy when choosing to use dMH platforms but are more inclined to engage if recommended by a trusted individual, including a provider or peer. However, some youth expressed a general resistance to any recommendations about dMH, valuing their own decisions about whether to use dMH. These contrasting findings suggest that eliciting youths’ consideration about using dMH must be explored to minimize the risk of rejection and resistance to dMH. Inquiries about dMH from trusted individuals may be followed by youth considering dMH.

Once committed to using a dMH platform, our findings suggest that a positive onboarding experience can facilitate positive user engagement. Youth participants in this study recommend that educational information about dMH and a collaborative approach to engaging youth is critical for increasing the continuous use of dMH. Similar to our findings, a 2023 scoping review of therapeutic dMH platforms for schizophrenia identified thorough initial training including, educational sessions and a hands-on support team as a predictor of user engagement levels [45]. To increase trust in reluctant youth, we recommend that dMH platforms facilitate a virtual orientation process that grants youth the opportunity to practice navigating through the platform and educates them about its intent, and security and privacy features.

Our findings indicated progress tracking features such as graphs and diaries could bolster user motivation and thus lead to higher user engagement. It has been found that increased insight into a user’s own personal health, along with empowerment, knowledge, and curiosity may increase engagement with the platform [25]. A research article postulates a psychological mechanism for how digital technologies use progress tracking to increase user motivation and engagement [46]. Another paper described the opportunities and challenges of passive and active progress tracking, arguing that the latter is more conducive to habit formation than a passive, automated one [47]. This suggests that active progress tracking, such as the diary feature suggested by participants , can be more effective in fostering user engagement than passive strategies such as data visualization. Some participants identified the potential for progress tracking to be discouraging for youth whose progress is nonlinear or in a crisis. However, although not contributing to user motivation, progress tracking features could bring youth’s attention to unconscious trends of improvement, decline, or stagnation in their mental health.

Our findings suggest an intuitive and accessible user interface is conducive to increased engagement with dMH platforms. Other literature supports our findings, including a qualitative study on the dMH platform Artemis; youth testers preferred a “simple, modern, and consistent” user design and “calming” color schemes instead of identifying technical issues as problematic and disruptive to user engagement [48]. User interface design, content quality, and personalization significantly influence youth engagement [49]. Visually engaging and user-friendly interfaces are crucial in encouraging youth to engage with digital health tools. Our findings indicated that youth preferred an application-based delivery over a web-browser-based dMH platform.

In our findings, participants emphasized dMH, including crisis resources such as a “chat now” feature. In a review of dMH applications’ features, a stark 1 out of 31 CBT applications targeting depression were identified that included explicit crisis support features, defined as prominent, identifiable, and intended for use during mental health crises. Such features were not interactive, including a detailed crisis plan or prominent links to suicide prevention hotlines or support centres. This limited presence of crisis support in existing dMH applications aligns with the youth’s call for such features on dMH platforms [47].

### Theme 3: Sustaining Engagement—Strategies for Optimum User Retention

Participants in our findings highlighted several factors that hindered their ability to consistently use dMH platforms, including the challenges of maintaining engagement, integrating usage into daily routines, and competing with other digital distractions. This decline in engagement over time reflects a common issue with adherence to digital health interventions [50-52]. To that end, designing platforms that offer ongoing value, such as personalized content, may help foster long-term use. Further, nonpersonalized interfaces can result in frustration and disengagement [13]. A systematic review by Opie et al [53] found that personalized feedback primarily delivered through a dMH internal messaging system from providers to youth was linked to a positive increase in user outcomes.

Participants also identified competition with social media as a barrier to using dMH, as youth often use social media to seek and maintain relationships for educational purposes and entertainment [54]. The immediate gratification and diversity of applications offered by social media can divert attention from health-focused applications. However, by incorporating these aspects, platforms promoting social connectedness enabled by social networks through peer support have been shown to increase engagement [25,53,55]. In contrast, research showed that integrating social media may affect the credibility of the dMH platform [53]. To compete, dMH platforms must offer a similarly compelling user experience. The increased face-to-face interactions facilitated by proper and consistent dMH use by providers can similarly engage youth users. Participants emphasized push notifications and dynamic visual and interactive content would optimize youth retention and engagement (Table 2). Consistent with existing research, participants emphasized the need for customizable, well-timed, relevant notifications that fit naturally into their daily routines [26,56]. In addition, the use of rewards or positive reinforcement to motivate individuals was mentioned within focus groups, which is consistent with previous research on persuasive design techniques [52,57]. However, collecting data for personalized notifications may impact users’ privacy without youths' informed consent. Research also suggests that the development of notification fatigue, where users feel overwhelmed by constant alerts, can lead to disengagement or deletion of the platform [58]. Similarly, while not youth-specific, notifications promoted the engagement of users who consistently use the platforms rather than inactive users [59].

Our findings can be situated within a Self-Determination Theory framework, which suggests that human behavior is motivated by 3 innate psychological needs: autonomy, competence, and relatedness [60]. Participants emphasized privacy controls and personalization, which align with Self-Determination Theory. Progress-tracking features and user-friendly design can increase youth’s sense of competence. The need for relatedness can be linked to the crisis support and provider communication features suggested by youth for dMH. The satisfaction of these psychological needs may facilitate engagement and long-term retention for youth on dMH.

### Practical Recommendations for dMH Implementation

Throughout focus groups, youth participants provided recommendations on how to implement and maintain dMH platforms, including building trust by using these platforms, features that promoted engagement and ways to maintain youth attention and engagement throughout dMH adoption. The themes outlined in this paper offered the ability to provide practical recommendations for implementing dMH platforms in clinical settings for youth and young adults dealing with mental health concerns. Table 2 details the recommendations based on our findings. It is important to note that dMH implementation relies on an individual’s access to an electronic device and the internet, which may be feasible in low-resource settings.

**Table 2.** Recommendations are based on participant focus groups.

Theme and recommendations		Description
Theme 1: Building Trust		
	Ensuring privacy, control, and security	Prioritize transparent data usage policies and emphasize the platform’s commitment to data security to foster trust among youth. Empower youth by offering customizable privacy settings that allow them to control who accesses their mental health information. Platforms should provide flexibility for users to selectively involve providers, parents, or guardians, ensuring youth needs are met. Build trust by clearly communicating privacy policies and data security protocols before onboarding. Highlight confidentiality without overemphasizing security features to avoid triggering concerns about privacy invasions. Strengthen data security measures to protect youth from the emotional impacts of potential data breaches and foster confidence in the platform’s safety.
Theme 2: Platform Features for Engagement		
	Streamlined, collaborative onboarding processes	Implement a comprehensive, step-by-step onboarding process hosted on the digital mental health (dMH) platform and engaging their provider. The onboarding process should be educational, explaining the purpose and key features of the platform to sustain user interest.
	Progress tracking and a user-friendly interface	Offer customizable tracking tools like graphs and diaries to help youth monitor their mental health and increase autonomy. Active tracking can foster engagement and support self-reflection. Platforms should emphasize that progress tracking is not just about linear improvement but also about understanding personal growth, even in times of crisis. Develop simple and intuitive platforms with customizable features. Allow youth to personalize notifications, communication settings, and resources to suit their needs and preferences. Mobile-based applications are preferred over a web platform for ease of access.
	Crisis support and professional communication	Include easily accessible crisis resources. Youth mentioned the importance of timely crisis interventions. Facilitating communication with professionals via the platform can provide an added layer of support, making the platform a go-to resource in times of need.
Theme 3: Sustaining Engagement		
	Guided dMH^a^ platforms	Ongoing human guidance and support can improve adherence by providing regular check-ins or nudges as well as personalized feedback from a professional to maintain motivation. Knowing that professional support is available can help youth build confidence when navigating dMH to promote sustained use. Regular interaction with a professional or trusted individual can promote a sense of accountability, leading to higher engagement rates as opposed to unguided approaches.

a dMH: digital mental health.

### Strengths and Limitations

The project’s strengths included involving youth from different geographic regions in rural and urban communities where Wi-Fi varied in accessibility. We recruited a diverse sample of youth with respect to geography, sector of involvement, gender, and age. Another important strength is that the focus groups were facilitated by a researcher, youth researcher partner, and implementation lead. A summary of the focus group was shared with participants within 24 hours to receive additional reflections and confirm the accuracy of what was captured from the focus groups.

There are several limitations worth noting. The focus groups were conducted during the COVID-19 pandemic, which may have influenced youths’ reflections about using online applications and e-tools via dMH. The increased usage of digital platforms during the pandemic period may have made participants more receptive to dMH compared to prepandemic conditions. The pandemic may have also heightened youth’s mental health concerns, influencing their expectations and needs from dMH. This could affect the generalizability of our findings in postpandemic settings. However, the research team probed broadly about the youths’ perceptions of dMH before the pandemic to obtain reflections that were not dependent on the context at the time. While the youth had a brief explanation and introduction to the platform before each focus group, all were conducted before youth were onboarded or using the dMH; consequently, the feedback about dMH was general rather than specific to a particular platform like Innowell. In addition, only Albertan youth participated in the focus groups, affecting the generalizability beyond our context.

### Conclusions

In this paper, youth perspectives on facilitators of the implementation of dMH perspectives were explored. In total, 3 major themes emerged through thematic analysis. The first, “Building Trust,” includes strong confidentiality and robust data security measures as crucial for youth trust in a dMH platform. Second, “Platform Features to Maximize Engagement” includes a thorough onboarding process, a simple and intuitive user interface, and progress tracking to engage with youth users. The third, “Sustaining Engagement,” centers on strategies like notifications and reward systems to increase the likelihood that users remain users of dMH platforms. The findings informed recommendations for developing dMH to increase youth uptake of dMH platforms, increase engagement with dMH platforms, and ensure that youth stay committed to dMH platforms.

## Abbreviations

dMH: digital mental health
